# Medical History from the Earliest Times

**Published:** 1892-07-23

**Authors:** E. T. Withington


### Medical History from the Earliest Times.

By E. T. Withington, M.A., M.B. Oxon.

### XVIII.—THE METHODIC SCHOOL.

Asclepiades had many followers, some of whom seem to have assumed his name in the hope of thereby acquiring some of his popularity, but they would probably have soon been absorbed by one or other of the dominant schools had not one of them in his old age conceived the idea of at once simplifying medicine and discovering a middle way which should avoid the errors and combine the excellences of the Empirics and Dogmatists. This was Themison, of Laodicea, whose fellow-citizens afterwards tried so unsuccessfully to find a middle way in religion, and who is probably referred to by Juvenal in the uncomplimentary line, "*Quot Themison ægros autumno occiderit uno!*"

Instead of searching for the causes of disease, like the Dogmatists, or distinguishing different disorders by their symptoms, like the Empirics, Themison declared that the physician should observe what symptoms various diseases have in common. He would then find that in all, or nearly all cases, there was an increase or diminution of the secretions and excretions, and adapting this to the theory of Asclepiades, Themison argued that all diseases were due to a constriction or a relaxation of the "pores." These were the two fundamental "communities" of the Methodists, but a third or "mixed" was soon added to include cases where one part of the body was relaxed and another constricted. The treatment followed at once on the principle "*contraria contrariis*," and consisted of astringents or laxatives, while in mixed communities the most threatening of the two states was to be counteracted. Among astringent remedies were cold air and water, vinegar, decoctions of various herbs, especially the plaintain, and the minerals alum, lead, and chalk, which were used externally. The great "laxative" was bleeding, whether by venæsection, cupping, or leeches (which last the Methodists introduced into European medicine), after which came poultices, fomentations, and warmth generally. Purgatives they entirely rejected, declaring that they only substituted the opposite form of disease.

The Methodists thus agreed with the Dogmatists that the physician might reason from the seen to the unseen, *e.g.*, from the state of the secretions to that of the pores, while, like the Empirics, they taught that diseases are to be judged from their symptoms, and not from their causes, and they differed from both in the doctrine that it is unnecessary to consider the individual peculiarities of a case when once classed under one of the "communities." On the whole, they were most nearly allied to the Empirics. Like them they despised anatomy, though it might be useful to know the names of parts. They held that most diseases were general, and that in any case the locality was unimportant, for constriction and relaxation are the same wherever they occur, and require the same treatment. Nor was it necessary to know the remote causes of disease, for they always act by producing one of the above conditions. Thus, like the Empirics, they reduced medicine to a system of treatment, and Themison's boast that he had reversed the aphorism of Hippocrates, and that life was long and the art short, was not unjustified. The stricter Methodists held that even in cases of poisoning it was not necessary to consider the poison, but only the state of constriction or relaxation produced by it, but in others common sense was not entirely absorbed by system, and to meet such cases they invented a fourth community, the "prophylactic," just as the Empirics had escaped from a similar difficulty by means of "epilogism."

The Methodic doctrines were brought to perfection by Thessalus of Tralles, who called himself "Conqueror of Physicians," claimed to be the inventor of the whole system, and offered to teach it anybody in six months. He flourished under Nero, and dedicated one of his works to that emperor, in which he declares that he has founded a new sect, the only true one, "for no preceding physicians have left anything profitable either for the observation or cure of disease." His principal contribution to Methodism was the "metasyncritic" or alterative mode of treatment, by which he pretended that the state of the whole body, and especially of the pores, might be entirely changed. Though he claimed this, like everything else, as his own invention, he might have found it, and probably did find it, in the Hippocratic writings, whence we may take the simplest and most convenient example. In chronic disorders, says the author of the treatise, "On Internal Diseases," it is often advantageous to try to make the patient fatter. For this purpose the amount of food should be gradually diminished and that of exercise increased, till the patient finally eats one-tenth the usual quantity and walks twelve miles or more daily. After maintaining this for a few days the conditions should be gradually reversed, the food increased, and the exercise diminished, till the normal state is regained. As appropriate diet for the first stage the writer recommends roast pork and sour wine, and for the second bread, porridge, fatty substances, and sweets. Here we have the alterative treatment of the Methodists, with its "resumptive" and "recorporative" cycles in the simplest form, and Thessalus merely added complicated notes of diet and certain drugs to which he attributed special "alterative" properties. "This method," says Galen, "became a sheet-anchor for all who could not make a diagnosis, and was often successful, for many diseases are due to errors in diet."

The later Methodists, while departing considerably from the strict rules of the sect, still retained the title, and it is especially applied to Soranus of Ephesus, who flourished shortly before Galen (about A.D. 100). Many fragments of his works have survived, especially from the treatise "On Diseases of Women," which was copied by another Methodist, Moschion, and it has been shown that the writings of Cælius Aurelianus are little more than Latin translations from Soranus. In them we find much attention paid to anatomy and diagnosis, matters despised by the orthodox Methodists, and the general rules of treatment, which always formed the strong point of the sect, are particularly well given. Thus, fresh air is said to be even more important than diet, for we are always breathing, but eat only at intervals, while charms and incantations, though they have no objective efficacy, are not to be entirely despised, for they may sustain the hopes and therefore the vitality of the patient.

We shall be able to trace a close analogy between Methodism and the Brunonian system, which flourished at the close of the last century, nor are there wanting resemblances and contrasts to a still existing "school" of medicine, and the system of Themison and Thessalus is in some respects the exact counterpart of that of Hahnemann. The term "allopathy" would indeed have puzzled Soranus, but if we take it as denoting a medical system of which the one therapeutic rule is "*Contraria contrariis curentur*," it may fairly be applied to the Methodic and Brunonian doctrines, and to them only. The Methodic system originated during a period of transition, and was rejected by the chief adherents of the older schools. Thus Celsus, who holds the balance so fairly between the Empirics and Dogmatists, has no good word for the Methodists, whom he compares to cow-doctors and savages, declaring that the old physicians knew all about the "communities," but were not content with them, and that the method of treating diseases in bulk is permissible only in large slave infirmaries, where nothing better can be got. Galen was their mortal enemy, and called them "the asses of Thessalus," though, as usual in such cases, it is hard to say which side first employed abusive language. Thessalus, like Hahnemann, appealed from the profession to the public, and, like Hahnemann, he was successful; crowds followed him, including, if we may trust a prejudiced witness, all the ne'er-do-wells in Rome. Nor are the causes of this success far to seek. Here was a theory suited by its connection with the dominant Epicurean doctrines to the philosophic Greek. Here was a rule of thumb which attracted the practical and methodic mind of the Roman. Above all, here was a short and easy system by which a self-confident individual might, with least preliminary labour, put money in his purse, label himself with an attractive name, and become a fashionable practitioner.

The fact that so many Methodists were distinguished as "ladies' doctors" may indicate that that sex was particularly attracted by the new system. Celsus tells us that it was found useful in slave infirmaries, and we may, perhaps, picture the Lady Bountiful of the period walking through such an institution, armed with the last pamphlet by Thessalus, and followed by a slave bearing the typical Methodic remedies, a bottle of leeches for all the "stricti" on one side of the ward, and a jar full of decoction of poppy heads and honey—the famous diacodion of Themison—for all the "laxati" on the other.

